# Traffic, Air Pollution, Minority and Socio-Economic Status: Addressing Inequities in Exposure and Risk

**DOI:** 10.3390/ijerph120505355

**Published:** 2015-05-19

**Authors:** Gregory C. Pratt, Monika L. Vadali, Dorian L. Kvale, Kristie M. Ellickson

**Affiliations:** 1 Environmental Analysis and Outcomes Division, Minnesota Pollution Control Agency, 520 Lafayette Road, St Paul, MN 55155, USA; E-Mails: monika.vadali@state.mn.us (M.L.V.); dorian.kvale@state.mn.us (D.L.K.); kristie.ellickson@state.mn.us (K.M.E.); 2 Division of Environmental Health Sciences, School of Public Health, University of Minnesota, 420 Delaware Street SE, Minneapolis, MN 55455, USA

**Keywords:** traffic, socio-economic status, air pollution risk, environmental justice

## Abstract

Higher levels of nearby traffic increase exposure to air pollution and adversely affect health outcomes. Populations with lower socio-economic status (SES) are particularly vulnerable to stressors like air pollution. We investigated cumulative exposures and risks from traffic and from MNRiskS-modeled air pollution in multiple source categories across demographic groups. Exposures and risks, especially from on-road sources, were higher than the mean for minorities and low SES populations and lower than the mean for white and high SES populations. Owning multiple vehicles and driving alone were linked to lower household exposures and risks. Those not owning a vehicle and walking or using transit had higher household exposures and risks. These results confirm for our study location that populations on the lower end of the socio-economic spectrum and minorities are disproportionately exposed to traffic and air pollution and at higher risk for adverse health outcomes. A major source of disparities appears to be the transportation infrastructure. Those outside the urban core had lower risks but drove more, while those living nearer the urban core tended to drive less but had higher exposures and risks from on-road sources. We suggest policy considerations for addressing these inequities.

## 1. Introduction

It has been known for at least 50 years that economic status can be a modifier of the effects of air pollution on health [1]. Since then numerous studies have shown a disproportionate exposure to traffic and air pollution among non-whites and populations with lower socio-economic status (SES) [2,3,4,5,6,7,8,9,10,11,12,13,14,15]. These disproportionate exposures have been linked to higher risks and greater incidence of a variety of health effects among non-white and lower SES populations [16,17,18,19,20,21,22,23,24,25,26,27,28,29,30,31,32,33,34,35,36,37].

Many of the above-cited studies used exposure metrics that were limited to a single pollutant and were not highly spatially resolved. Those metrics included modeled county, zip-code, or census tract level estimates of air pollution concentrations, measurements at the nearest monitoring site (often widely spaced), proximity to air pollution point sources, and proximity to high traffic corridors, among others. Such broad-based measures of exposure may not capture the geospatial detail and complexity of pollutant concentrations. In addition, estimates of exposure to a single air pollutant may not capture the integrated chemical stresses from cumulative exposure.

The relationship between SES, air pollution and health effects is complex and confounded by other stressors. Economically disadvantaged groups tend to be exposed to more family turmoil, violence, instability, crowding, and noise. Neighborhoods with high levels of poverty tend to have higher crime rates, more substandard housing and offer poorer schools and municipal services [3]. The accumulation of multiple environmental stressors may increase vulnerability to a particular individual stress such as air pollution from traffic.

A single demographic datum representing a geographic unit, such as median household income, may not capture the variability within the unit, which may lead to misclassification errors, especially if exposure or health data are available with greater precision, e.g., at the individual level. Similarly, there are misclassifications in exposure when taken at aggregated geographic units. Despite the potential misclassification errors, there is a large body of research demonstrating significant links between exposure and SES e.g., [1,2,3,4,5,6,7,8,9,10,11,12,13,14,15,16,17,18,19,20,21,22,23,24,25,26,27,28,29,30,31,32,33,34,35,36,37] suggesting that the relationships are strong. Given these potential sources of error, it is important to work at the most geospatially resolved scale as possible. We chose to work at the spatial scale of the smallest census unit with enumerated SES data, the block group, typically with 600−3000 residents.

Minnesota is an interesting case study because it includes a major metropolitan area, extensive agricultural land, and undeveloped rural and wilderness areas. The State population is relatively homogeneous (81.6 percent Non-Hispanic White in the 2010 census) compared to many other states, but it also is home to several Native American reservations and has the largest urban Native American population in the US. Recent immigration, notably Hmong and Somali, has increased diversity in the Minneapolis-St. Paul metropolitan area. The metropolitan area is defined in two ways. The US Census Bureau defines the metropolitan statistical area as 11 counties in Minnesota and two in adjacent Wisconsin. In contrast, the metropolitan planning organization, the Metropolitan Council, is granted regional authority powers in state statutes for the seven counties at the core of the metropolitan statistical area. Metro Transit, the transportation branch of the Metropolitan Council, operates bus and rail transit services in the seven county metropolitan area. Our analyses focus on the seven county metropolitan area (Metro Area).

The metropolitan statistical area was 16th largest in the 2010 census with a population of 3.34 million distributed among 182 cities and townships. It ranks as the 74th most sprawling metropolitan area in rankings of 261 metropolitan areas [38], and has the nation’s eighth largest highway system per capita [39]. Despite these characteristics, the Metro Area ranks high for active lifestyles [40] and has been recognized for its bicycle infrastructure and bicycle friendliness [41]. Air pollution levels are generally low for its size due to consistent northwesterly wind flow that brings clean air and carries away pollutants efficiently. Although the Metro Area ranks high on measures of health, education, safety, and civic engagement, some have noted a “Minnesota Paradox.” Despite the generally high quality of life in the Metro Area, disparities among racial groups in economic and social measures are among the worst in the nation [42,43].

We developed two measures of exposure that are highly resolved in space: traffic density [44] developed on a 50 m resolution grid across the state of Minnesota, and the MNRiskS model [45] that estimates concentrations and risks at 60,613 receptor locations in the state from 235 air pollutants emitted from all inventoried sources (including point, non-point, and mobile sources). The spatial resolution of these data is much greater than the resolution of available SES data, *i.e.*, census block groups. We therefore calculated means of traffic density and MNRiskS estimates at the block group level in order to investigate relationships. Our work extends previous studies relating environmental impacts to SES by: (1) exploring features of SES (like housing and transportation) that have not been previously examined at this level of detail; (2) working in a northern US metropolitan area that is racially and economically homogenous; (3) using highly spatially resolved estimates of exposure and risk; and (4) evaluating impacts by subcategories of sources as well as cumulative impacts across all sources and 235 pollutants.

## 2. Experimental Section 

The American Community Survey 5-Year Summary File 2010 was downloaded from the US Census Bureau at the block group level. In addition to the variables available in the tables, we computed several additional variables including fraction non-white, vehicles per household, fractions with income above and below $60,000, fraction with home value above $250,000, and others (see Figure S1 in the Supplementary Material). Those who self-identified as having Hispanic ethnicity (either alone or in combination with another racial group) were taken as a separate demographic group and were excluded from other racial groups in this analysis. We included all racial minorities and Hispanics in the group identified as “nonwhite.”

Given the wide variability in total population across block groups, we calculated each population group as a fraction. For example, the white non-Hispanic population in each block group was divided by the total block group population to give the fraction of white non-Hispanics in the block group. Most of the variables had a skewed distribution, often approaching log-normal. Therefore, all data were log-transformed (after adding 1 due to zero values) and then normalized by subtracting the mean and dividing by the standard deviation. This transformation resulted in the variables scaling similarly, aiding the interpretation of the regression results. Statistical analyses used R 3.1.0. 

An earlier version of the MNRiskS modeling system based on 2002 emissions was described previously [45]. It has been improved and updated with new emissions data every three years since its initial development using 1999 emissions. The 2008 version was used here. Briefly, the system consists of three components: an emissions inventory, air dispersion/deposition modeling, and risk estimation. Air emissions of 235 pollutants were included from all inventoried point sources (12,271 processes), on-road mobile sources, 12 subcategories of non-road mobile sources, and 18 subcategories of area (non-point) sources (see Table S1 in the Supplementary Material for subcategory details). 

The air dispersion and deposition modeling component of MNRiskS used the EPA regulatory model AERMOD. Point sources were modeled using available stack parameter data (fugitive sources were treated as volume sources). On-road mobile sources in high traffic corridors in the Metro Area were modeled as 100 meter volume sources located adjacent to one another every 100 meters along the roadways. Other on-road mobile source emissions, as well as the non-road and non-point (area) emissions were apportioned to census block groups and modeled as polygon area sources simplified to no more than 30 vertices. Apportioning to block groups was done using surrogates for each subcategory (see Table S2 in the Supplementary Material for details on the surrogates). Air concentrations and deposition were calculated at 60,613 receptor points comprised of a 300 m grid in the core of the Metro Area, a 3 km grid outside the core, each block group centroid, and the locations of maximum concentration from each point source. 

The air dispersion modeling results were used as inputs to risk modeling following the United States Environmental Protection Agency (US EPA) Human Health Risk Assessment Protocol (HHRAP) methodology [46]. Adult resident cancer risks and non-cancer hazard indices resulting from all pollutants were extracted at each receptor by source category (point, on-road mobile, non-road mobile, and area/non-point). The cancer risks modeled in the MNRiskS system represent the increased chance of developing cancer due to a lifetime exposure at the model-estimated rates to all of the substances with cancer toxicity values. Since demographic data were available at the block group level, risk metrics were calculated by block group. Mean, median, and maximum risks were highly correlated—we chose to use mean risks.

Traffic density was calculated using 2005 traffic count data. The methods were described previously [44]. Briefly, a kernel density algorithm was applied to traffic count data available from the Minnesota Department of Transportation for every roadway segment in the state. The density calculation generated a 50 m resolution raster surface. The density value in each cell reflects all traffic on all roads within the distance specified in the kernel density algorithm. The effect of a given roadway on the raster cell value depends on the amount of traffic on the road segment, its distance from the raster cell, and the form of the algorithm. We used a Gaussian algorithm in which traffic influence became insignificant beyond 300 m. Block group mean traffic density was calculated for comparison with the demographic data. 

We use the term exposure to apply to both traffic and air pollution and the term risk to apply to the estimated risks to a Resident adult (as defined in HHRAP). The risks were calculated as the sum of risks over all pollutants in MNRiskS having a chronic toxicity endpoint, either cancer or non-cancer (see Table S3 in the Supplementary Material for a list of pollutants and toxicity values). Acute risks were not addressed in this study.

## 3. Results and Discussion

### 3.1. Study Area 

Table 1 gives a demographic breakdown of the population examined in this study. The total population in Minnesota is fairly evenly divided between the Metro Area (54%) and outside the Metro Area (called outstate or non-metro, 46%). However, most minorities (e.g., 86% of Asians, 87% of Blacks, and 67% of Hispanics) reside in the Metro Area, with the exception of Native Americans, 69% of whom reside outstate, often on tribal land. Overall, 75% of non-whites and 49% of whites reside in the Metro Area. Income, home values, rents, and education levels are generally higher in the Metro Area. The Metro Area population is also younger, more likely to commute by walking or transit, and particularly in the urban core, has a lower rate of vehicle ownership.

**Table 1:** Demographic breakdown of the population of the seven county Minneapolis-St. Paul metropolitan area, areas outside the metro, and the entire state.

Demographic	Metro	Non-Metro	State
Block Groups	2085	2026	4111
Total Population	2,807,902	2,434,012	5,241,914
Asian	173,813	29,255	203,068
Black	221,266	32,027	253,293
Hispanic	156,071	77,645	233,716
American Indian	16,109	36,098	52,207
NonWhite	636,174	208,899	845,073
White	2,171,728	2,225,113	4,396,841
Less than High School	136,915	162,331	299,246
Bachelors Degree	488,704	247,716	736,420
House Value > $250K	365,283	164,770	530,053
Owner Occupied Housing	790,821	757,306	1,548,127
Rent < $700	84,010	113,370	197,380
Rent > 30% Income	152,194	94,975	247,169
Rental Housing	319,896	217,894	537,790
>1 Vehicles in Household	662,678	651,681	1,314,359
Commute by Walk/Transit	112,334	59,116	171,450
Drove Alone	1,138,275	942,865	2,081,140
No Vehicles in Household	87,946	56,296	144,242
Below 100% of Poverty	276,096	266,037	542,133
Below 150% of Poverty	448,882	461,489	910,371
HH Income > $60K	829,383	642,501	1,471,884
Children Under 10	383,234	315,404	698,638
Over 65	292,138	366,025	658,163

The division between the Metro Area and outstate is imperfect. There are parts of the geographic area defined as “Metro” in this study that are rural and agricultural, and there are urbanized areas outstate. However, given that the seven county Metro Area is governed as a discrete entity, we decided to focus our analysis there (results for the whole state and for outstate areas are discussed and presented in the Supplementary Material). There is also more diversity in the Metro Area across nearly all of the demographics, which enables easier detection of relationships that may exist across demographic groups.

### 3.2. Correlations 

**Figure 1:**
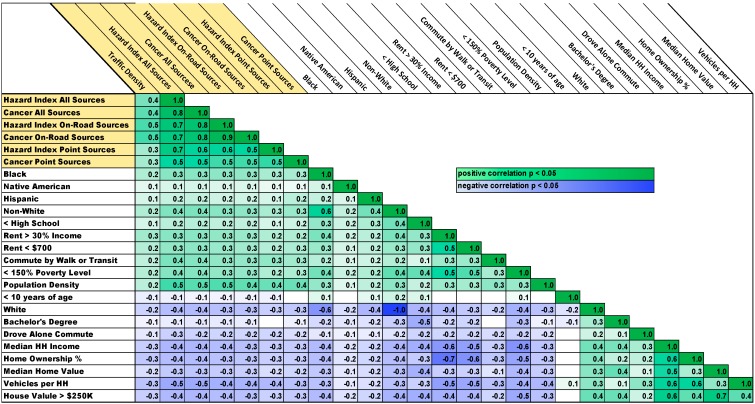
Kendall’s Tau-B nonparametric correlations for 2085 block groups in the Metro Area. The first seven rows and columns are variables describing air pollution exposure and risk. The remaining columns are demographic variables. All reported coefficients are statistically significant with *p* < 0.05 for the lightest shading and decreasing to *p* < 0.00001 for the heaviest shading.

Our first step in investigating relationships between demographics and exposure to traffic and air pollution was to look at correlations (Figure 1). Metro Area correlations followed a clear pattern in which traffic exposure and air pollution risks from all sources were positively correlated with minorities, measures of low SES, and population density. In contrast, exposure and risk were negatively correlated with whites and those with higher SES. This pattern of relationships was also found outstate and for the entire state (see Figures S2 and S3 in the Supplementary Material), but the relationships were not as strong or consistent across all categories.

**Figure 2:**
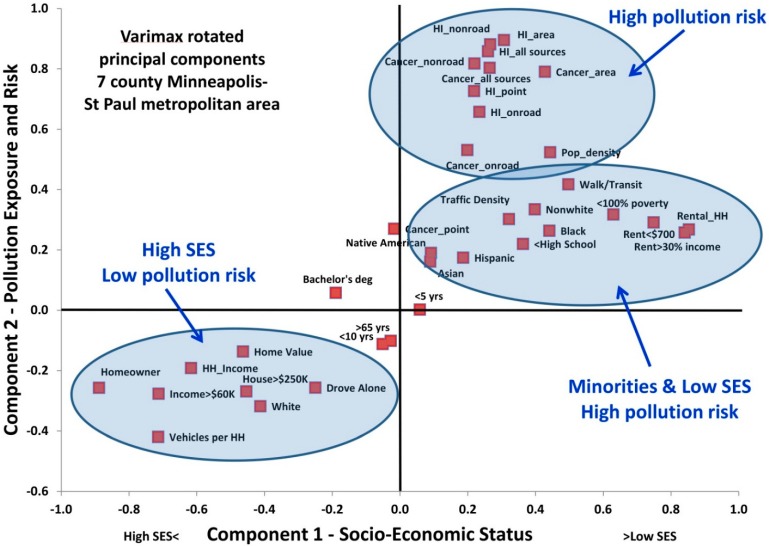
Factor loading plot for the first two components identified in the principal components analysis. The prefix HI refers to non-cancer hazard index.

The pattern found in the correlations was confirmed by principal components analysis (PCA, Figure 2). The first two components identified by PCA explained 43% and 10% of the variance and were interpreted as relating to SES and to traffic/pollution exposure and risk, respectively. Five other components with eigenvalues > 1 explained minimal amounts of the variance. All of the variables describing exposure and risk loaded together for these two components, although cancer risk from point sources did not group as neatly as the other variables. Variables representing high SES and whites also grouped together in PCA, and variables representing non-whites and low SES loaded together. The cancer risk from point sources was the only risk variable that loaded negatively on the SES component, suggesting that point source impacts are more weakly related to SES than impacts from other source groups. 

**Figure 3:**
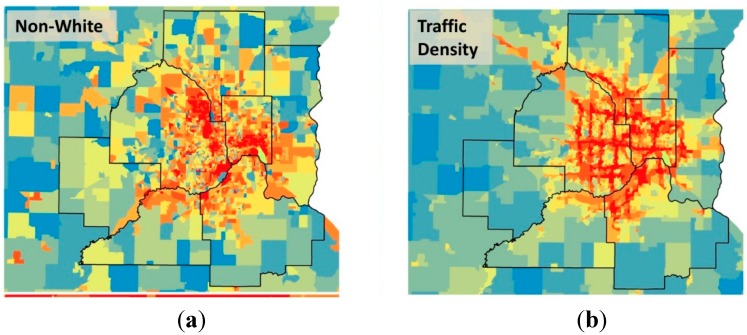

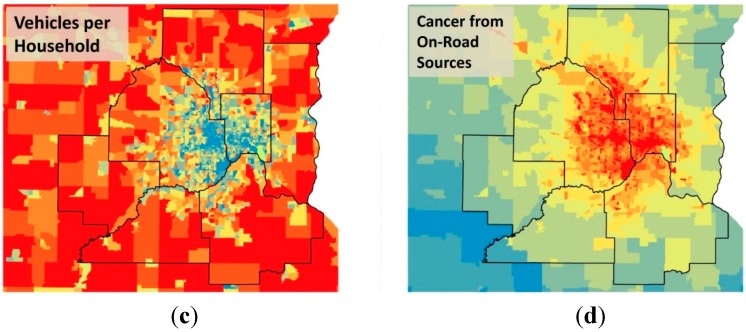
The Minneapolis-St. Paul Metropolitan Area with block groups colored according to (**a**) the fraction non-white population, (**b**) traffic density, (**c**) vehicles per household, and (**d**) cancer risk from on-road sources. The color palette in all four maps increases from blue to red by decile of the metric. The seven counties in the Metro Area are shown in bold outline.

Figure 3 shows the relationships between four of the variables geospatially. Viewing the data in this way shows clearly that non-white, traffic density, and cancer from on-road mobile sources are positively related with one-another, and that these three variables are negatively related to the number of vehicles per household.

### 3.3. Regressions and Insights into Relationships

Correlations and PCA begin to illustrate relationship patterns, but since many of the variables are highly correlated with one another, these analyses do not address multicollinearity issues and do not necessarily highlight the relationships that are most relevant. We used several multiple linear regression approaches to narrow the scope of important variables, including stepwise addition or subtraction of variables, entering all variables, looking at all combinations of subsets of variables up to ten variables at a time, and using the variance inflation factor (VIF) to remove variables. Adding more than six to eight variables usually did not improve the model as measured by the adjusted R^2^ values, the Bayesian Information Criterion (BIC), or the conceptual predictive statistic (Cp) (for example, see Figures S4 and S5 in the Supplementary Material). We took each exposure and risk variable in turn as the dependent variable and ran regressions using the demographic variables as independent variables. 

**Figure 4:**
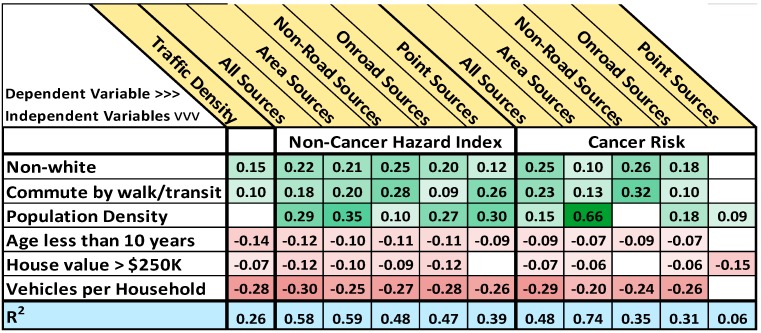
Each column represents one regression equation with the dependent variable at the top of the column and the independent variables listed down the side in column one. The reported values are the statistically significant (*p* < 0.05) regression coefficients with R^2^ values in the bottom row. Blank cells were insignificant in the model. Positive coefficients are highlighted in green and negative coefficients in red with *p* < 0.05 for the lightest shading to *p* < 0.0001 for the darkest shading.

After synthesizing the results from all of the regression approaches, we selected six variables that illustrated the strongest and most consistent relationships across the range of regressions (Figure 4 and Figure S6). The pattern in the results clearly shows that non-whites, non-drivers, and population density were positively related to exposure and risk while vehicles per household, high home value, and ages less than 10 years were negatively related. The VIFs for these variables ranged from 1.1 to 1.8 indicating that there is little collinearity among them. The 95% confidence intervals for the coefficients in the regression models are given in Figure S7 in the Supplementary Material.

We found that non-white was the best variable relating racial and ethnic status to exposure and risk. After adding non-white to the regression models, the variables for individual non-white population groups nearly always became insignificant. 

Two of the variables resulting from the regression synthesis represented transportation-related demographics (commuting by walking or transit and vehicles per household). After controlling for these two variables, the other variables relating to transportation usually became insignificant. The number of vehicles per household is greater outside the urban core. The number of vehicles per household appears to be a marker for living away from sources of air pollution since living away from population centers requires reliance on motor vehicles for transportation. Commuting by walking or transit is a marker for living in the urban core where distances to destinations are shorter and transit is more readily available. In addition, the costs of vehicle ownership may be prohibitive for lower income households.

Population density is a proxy for the urban core where air pollution risks are usually highest, and it was nearly always positively related to air pollution exposure and risk. However, population density was only weakly associated with other SES variables and with air pollution exposure and risk. One reason may be that there are areas of dense housing both in the urban core and in some outlying suburban areas. We note the apparent contradiction that population density was significantly related to risks from on-road mobile sources but not to traffic density. The regression models revealed that after controlling for the number of vehicles per household, population density became insignificant in the model for traffic density. In contrast, when risks from on-road mobile sources were the dependent variables, population density was significantly related, and vehicles per household, while still significant, was not as strongly related. We also note that population density was strongly related to the cancer risk from area sources. 

The population of children less than 10 years of age was consistently negatively related to air pollution exposure and risk in the regression analyses despite the fact that individual correlations were relatively weak. In addition, the children less than 10 years variable was not well correlated with many of the other demographic variables. This finding may indicate that there is information in the under 10 age demographic that is not contained in the other variables, perhaps that families with children under 10 years of age tend to locate in areas away from air pollution. 

We explored age-related variables geospatially and found that in the Metro Area the less than 10 years and 5−10 years groups were found disproportionately in the outer suburbs. In contrast, the under-5 age group was more likely to be found in the urban core. One hypothesis is that parents may move away from the urban core to suburban areas seeking more space and better schools when their children reach school age. The under-5 age group was a significant independent variable with a positive coefficient in some regression models. Other age-related demographics like seniors and women of child-bearing age were usually not significantly related to traffic and air pollution exposure and risk.

High home value was negatively related to exposure and risk. Although high-value homes are found throughout the Metro Area, including in the dense urban core, they appear to be preferentially located away from traffic and sources of air pollution, or alternatively, sources of air pollution have not been allowed near areas of high value housing.

In general, stronger relationships were found for the non-cancer endpoints than for cancer risk or traffic density. Cancer risks from point sources had weak associations with SES variables. Historically, many studies looked at the proximity of point sources of air pollution to vulnerable demographic groups and inferred relationships [47,48,49,50]. Our results agree with recent studies [5,6,7,8,14,32] showing that while point source emissions are related to race and SES, non-point source categories are more strongly related.

Three results stood out in our analysis. First, the regression analyses identified important variables (Figure 4) that were not always initially the most highly correlated. There is overlapping information in many of the demographic variables, and controlling for one effect may simultaneously control for the effects of others. Second, direct measures of income and poverty were less strongly related to exposure and risk than indirect measures like housing, age, and transportation metrics. Third, the metric of vehicles per household was consistently strongly and negatively related to all exposure and risk variables. 

Taken in sum we interpret these results as demonstrating statistically significant relationships between demographic factors and traffic and air pollution exposure and risk in the Metro Area. There were many statistically significant regression models relating exposure and risk metrics to demographic variables. Given the strong multicollinearity, it was not surprising that accounting for one variable changed the effect of others. The most important demographic variables were sometimes different for different dependent exposure and risk variables. However, we were able to select a set of independent variables that were consistently important across all of the dependent variables. We infer from our results that race, income, housing, education, age, and transportation utilization are all significantly related to traffic exposure and air pollution risk to varying degrees.

### 3.4. Risks by Demography and Air Pollution Source Category

Given statistically significant relationships between demographic groups and traffic and air pollution exposure and risk, we calculated mean exposures and risks for specific groups to investigate potential disparities using the following equation:

(1) $$\mathrm{demographic}\;\mathrm{group}\;\mathrm{mean}=\frac{\sum_{i=1}^{n}\left(demographic\;group\;total\;in\;BGi*\;env\;condition\;in\;BGi\right)}{\sum_{i=1}^{n}\left(demographic\;group\;total\;in\;BGi\right)}$$

where BGi refers to the block groups indexed from 1 to the total number of block groups (e.g., 2085 for the Metro Area).

**Figure 5:**
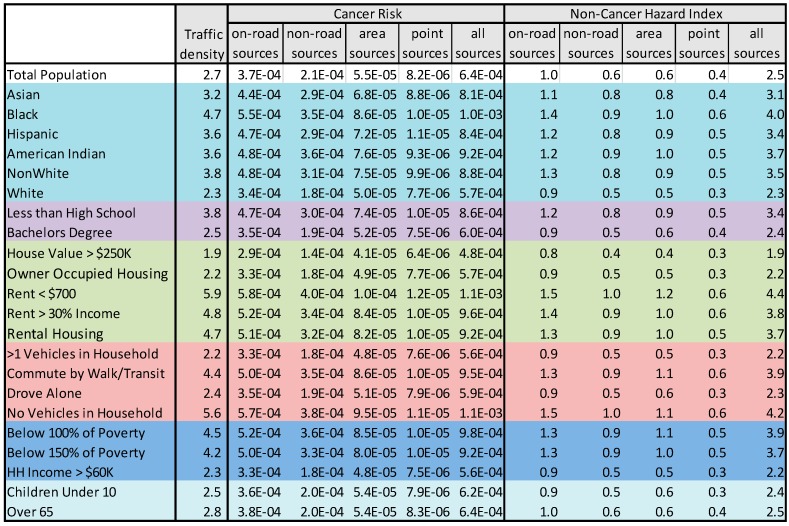
Breakdown of the Metro Area traffic density, cancer risks, and non-cancer hazard indices by demographic group and pollution source category.

**Figure 6:**
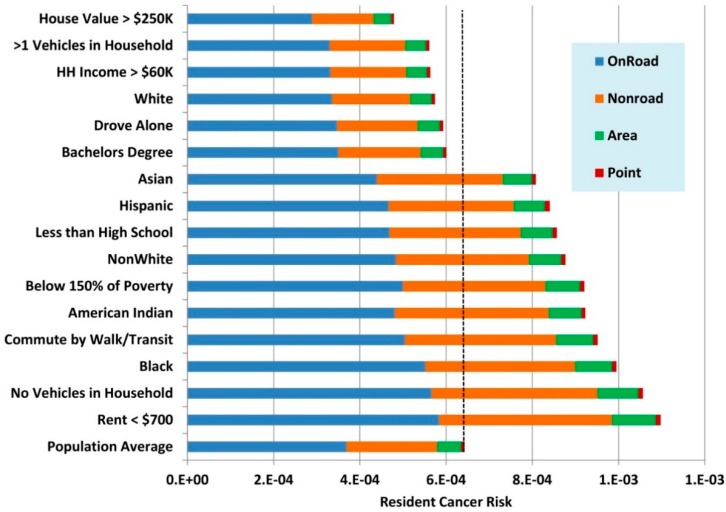
Estimated resident cancer risks from the MNRiskS system by major source groups for selected demographic groups in the Minneapolis-St. Paul metropolitan area.

**Figure 7:**
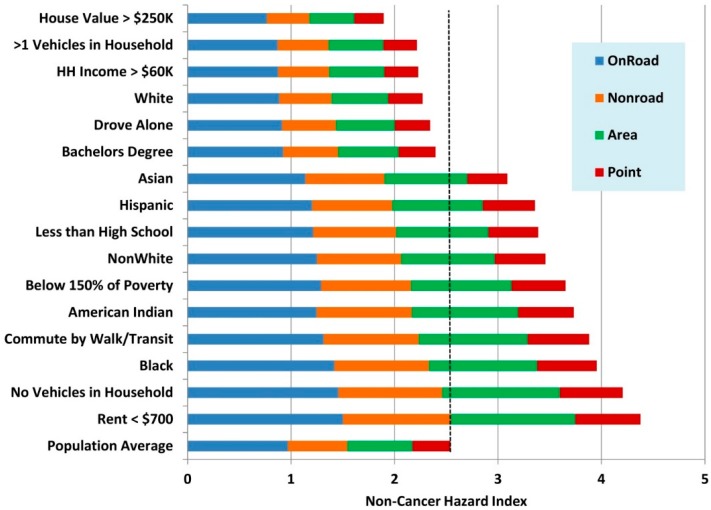
Estimated resident non-cancer hazard indices from the MNRiskS system by major source groups for selected demographic groups in the Minneapolis-St. Paul metropolitan area.

Figure 5 shows that the estimated exposures and risks for different groups in the Metro Area. Two subsets of these data are shown graphically in Figure 6 and Figure 7. The differences among demographic groups are clearly demarcated. Whites and high SES groups have lower traffic exposure and lower risk from air pollution from all sources. In contrast, exposure and risk were systematically higher than the mean for all non-white population groups and lower SES groups. Similar patterns were seen statewide (Figure S8 in the Supplementary Material).

Across all demographic groups, on-road mobile sources contributed the highest cancer risks, followed by non-road mobile sources and area sources. Point sources contributed a very small percentage of the cancer risk across all groups. Cancer risks were driven by diesel particles, which are emitted in all source categories. The high cancer risks from on-road mobile sources suggest that emissions from this source category are more likely to affect locations where people are living. 

Non-cancer hazard indices were more evenly divided among source categories across all demographic groups. However, on-road mobile sources usually contributed the greatest to the hazard index and point sources contributed the least for all groups.

Other studies [5,6,7,8,9,10,11,24] have shown similar disparities among demographic groups in traffic and air pollution exposure. Our contribution is to demonstrate disparities using cumulative and comprehensive metrics that are highly spatially resolved in a location that has not previously been studied—a location which is relatively unpolluted compared to similar sized metropolitan areas and which is relatively homogeneous in demography. 

### 3.5. Policy Implications

Our results highlight the environmental equity aspects of the transportation infrastructure. Low SES and non-white populations tend to own fewer cars, drive less, and commute by walking or transit. Other work has shown that poor tend to live close to city centers in large part because of better access to public transportation [51]. Despite driving less, low SES and non-whites bear disproportionately high air pollution impacts from all sources—especially transportation sources. In contrast, both white and high SES populations tend to have higher rates of car ownership and to drive more while the air pollution impacts at their homes tend to be lower. 

The build-out of transportation systems facilitating motor vehicles has allowed sprawling development that contributes to the environmental inequities we describe. This infrastructure has roots in the history of social injustice and the legacy of decision-making in which transportation arteries were located in areas with low land values that were often occupied by minorities and low SES populations. In turn, the presence of busy roadways may constrain improvement of low SES neighborhoods.

Addressing this legacy is a complex, long-term proposition. Our results support future decisions to: (1) reduce exposures from the current infrastructure; (2) recommend personal actions to reduce individual exposures; (3) encourage less polluting transportation technologies; and (4) build future infrastructure informed by environmental equity considerations. Impacts from the current infrastructure can be reduced with barriers between busy corridors and residential areas, traffic calming, optimized signalization, traffic circles *versus* stoplights and stop signs, implementing disincentives to driving, giving preferences to other transportation modes, and similar measures. Personal actions might include choosing to walk or bicycle as far from the traffic as possible, increasing the distance to nearby vehicles while driving or idling, considering traffic impacts in housing choice, and opting to drive less. Less polluting technologies that might be considered in future development include greater reliance on public transportation and providing incentives for electric and hybrid vehicles, although new vehicles may be less affordable to low SES groups most affected by transportation impacts. 

### 3.6. Limitations 

The most salient limitation in this analysis is that the frame of reference is households and individuals in a block group. Using this geographic scale leads to uncertainties in the demographic estimates and in the estimates of traffic and air pollution exposure and risk. The uncertainties in census data, especially at more resolved geospatial levels such as the block group, are well-documented [52]. For example, racial and ethnic minorities and undocumented persons tend to be systematically undercounted [53]. We do not know the full potential effects of these uncertainties on our analysis, but assuming that uncounted minorities tend to reside in locations similar to counted minorities, the likely effect of minority undercounting would be that we have underestimated disparities.

The exposures and risks are estimates of conditions at a particular location which are then averaged at the block group geographic scale. They do not represent an individual’s actual exposures and risks. In reality, a person’s exposure (and resulting risk) is the integration of time spent in multiple locations—indoors and outdoors—as that person moves through daily life. Thus our estimates of exposure and risk apply to a location rather than to an individual.

The potential exposure misclassification at various spatial scales was evaluated by Batterman *et al*. using concentrations of nitrogen oxides (NOx) from on-road mobile sources [54]. They estimated misclassification ranging from 20% to 40% going from household level parcels to census blocks and up to 80% going from parcels to census tracts. Block groups are intermediate in size between blocks and tracts, and the potential misclassification errors for block groups are expected to be intermediate between those for blocks and tracts. They also found that larger aggregations like census tracts and zip codes tended to overestimate NOx compared to parcels because they included concentrations on roadways while parcels are not located at these points of highest concentration.

Our estimates of traffic density and pollution risks have important uncertainties that have been discussed in previous work [44,45]. Cancer risks were the sum of risks from the 119 pollutants with cancer inhalation reference concentrations or oral slope factors. The risk driver pollutant in our cancer risk estimates was usually diesel particles. We used the toxicity value of 5 µg/m^3^ for diesel particles from the California Office of Environmental Health Hazard Assessment. This value has not been adopted by USEPA or the Minnesota Department of Health. In addition, the US allowable sulfur content of diesel fuel was lowered in 2006, and diesel particle control technology was mandated for new vehicles in 2007. As a result of these regulations diesel exhaust has become cleaner with time. A recent report from the Health Effects Institute [55] showed that particles from newer diesel engines were less toxic and did not cause cancer in rats. Taking account of the fleet penetration of new diesel technology could be expected to lower cancer risk estimates from on-road sources.

## 4. Conclusions 

Populations on the lower end of the socio-economic spectrum and minorities were disproportionately exposed to traffic and air pollution and at a disproportionately higher risk for adverse health outcomes. Despite driving less, the air pollution impacts were higher from all sources—especially transportation sources—at non-white and low SES households that tended to be closer to the urban core. In contrast, block groups with more white and higher SES populations, often located outside the urban core, tended to have higher rates of car ownership and to drive more while the air pollution impacts at their homes tended to be lower from all sources. Recognizing these inequities can inform decision-making to reduce them.

## Supplementary Files

Supplementary File 1
