# Supplementary material for: Traffic, Air Pollution, Minority and Socio-Economic Status: Addressing Inequities in Exposure and Risk
Source: Int J Environ Res Public Health. 2015 May 19;12(5):5355–72. doi: 10.3390/ijerph120505355 (PMC4454972; doi:10.3390/ijerph120505355)
Supplement: Supplementary File 1 [file ijerph-12-05355-s001.pdf]

Supplementary Information

Traffic, Air Pollution, Minority and Socio-Economic Status: Addressing Inequities in Exposure and Risk

---

Variable	Source
Mean Traffic Density	Traffic Density Model
Hazard Index All Sources	MNRiskS
Cancer All Sources	MNRiskS
Hazard Index On-Road Sources	MNRiskS
Cancer On-Road Sources	MNRiskS
Hazard Index Non-Road Sources	MNRiskS
Cancer Non-Road Sources	MNRiskS
Hazard Index Area Sources	MNRiskS
Cancer Area Sources	MNRiskS
Hazard Index Point Sources	MNRiskS
Cancer Point Sources	MNRiskS
Population Density	Decennial Census
Asian NonHispanic	Decennial Census
Black NonHispanic	Decennial Census
Hispanic	Decennial Census
Native American NonHispanic	Decennial Census
Non-White	Decennial Census
White NonHispanic	Decennial Census
< High School	American Community Survey
Bachelor's Degree	American Community Survey
Home Ownership %	American Community Survey
House Valule > $250K	American Community Survey
Median Home Value	American Community Survey
Owner Occuppied Housing	American Community Survey
Rent < $700	American Community Survey
Rent > 30% Income	American Community Survey
Renter Occupied Household	American Community Survey
> 1 Vehicle per HH	American Community Survey
Commute by Walk or Transit	American Community Survey
Drove Alone Commute	American Community Survey
No Vehicles in HH	American Community Survey
Vehicles per HH	American Community Survey
< 100% Poverty Level	American Community Survey
< 150% Poverty Level	American Community Survey
HH Income < $60K	American Community Survey
HH Income > $60K	American Community Survey
Median HH Income	American Community Survey
Children Under 10	American Community Survey
Children Under 5	American Community Survey
Female 20 to 39	American Community Survey

Figure S1. Variables explored in analyses.

Table S1. Non-point source categories and subcategories included in MNRiskS 2008.

**On-Road Mobile Sources**	**Non-Road Mobile Sources**	**Area (Nonpoint) Sources**
Cars	Airports	Feedlots
Buses	Commercial Marine Vessels	Industrial, Commercial, and Institutional Boilers
Trucks	Pleasure Craft	Composting
(diesel and gasoline)	Rail Yards	Animal Cremation
	Rail Operations	Food & Kindred Products
	Agricultural Equipment	Health Services
	Recreational Equipment	Miscellaneous Roadway Emissions
	Lawn & Garden Equipment	Miscellaneous Agriculture & Pesticide Emissions
	Construction & Mining Equipment	Non-industrial Consumer & Commercial Activities
	Industrial Equipment	Open Burning
	Commercial Equipment	Petroleum & Products Storage & Transport
	Logging Equipment	Residential Fuel Combustion
		Residential Wood Combustion—Outdoor Wood Boilers
		Residential Wood Combustion Indoor
		Residential Wood Combustion—Outdoor Recreation
		Surface Coating
		Agricultural Fires
		Wildfires & Prescribed

Table S2. Surrogates for apportioning non-point emissions to block groups.

**Area—Industrial + Commercial/Institutional**	**Commercial/Industrial Land Use in Metro—Outstate Land Use Categories 23,24 (Med. & High Intensity Developed)**
area—composting & animal cremation	land area
area—misc by population	population
area—food and kindred products	commercial land use in metro—outstate LU categories 23, 24 (med & high intensity developed)
area—health services	Block groups where hospitals located from ReferenceUSA
area—misc roadway by VKT	Vehicle Kilometers Travelled (VKT)
area—misc ag/pesticide	fraction of land in land use category 82 (crop production)
area—misc non-industrial: consumer & commercial	Households
area—open burning—yard & land	land area minus developed land area
area—open burning—household waste	Households in undeveloped land (all categories except 21,22,23,24)
area—petroleum & product storage & transport	gas station sales in BG from ReferenceUSA
area—residential fuel combustion	Households
area—residential wood combustion indoor	Households
area—residential wood combustion outdoor wood boiler	Households in undeveloped land (all categories except 21,22,23,24)
area—residential wood combustion outdoor recreation	Households
area—surface coating	commercial/industrial in metro—outstate land use categories 23,24 (med & high intensity developed)
area—Ag Fires	land in land use categories 71 & 81 (Grassland/herbaceous, Pasture/Hay)
area—Wildfires & Prescribed Forest Burning	model as individual volume sources
nonroad—commercial marine vessels	EPA major waterways shapefile apportioned to block groups
nonroad—Pleasure Craft	Water surface area
nonroad—railyards	model as volume sources located at coordinates
nonroad—rail	railroads shapefile with weighting for activity
nonroad—Agricultural Equipment	land in ag uses (land use categories 71, 81, 82)
nonroad—Off-highway Vehicle Recreational Equipment	land area
nonroad—Off-highway Vehicle—Lawn & Garden Equipment	Households
nonroad—Off-highway Vehicle—Construction & Mining Equipment	med&hi intensity developed outstate—combine construction + industrial/commercial in metro

Table S2. *Cont.*

**Area—Industrial + Commercial/Institutional**	**Commercial/Industrial Land Use in Metro—Outstate Land Use Categories 23,24 (Med. & High Intensity Developed)**
nonroad—Off-highway Vehicle—Industrial Equipment	commercial/industrial in metro—outstate land use categories 23,24 (med & high intensity developed)
nonroad—Off-highway Vehicle Commercial Equipment	commercial/industrial in metro—outstate land use categories 23,24 (med & high intensity developed)
nonroad—Off-highway Vehicle—Logging Equipment	land use categories forest = 41, 42, 43, 52
onroad—lower traffic segments—gas	VKT
onroad—lower traffic segments—diesel	diesel VKT
onroad—high traffic segments—gas	model as individual volume sources (pseudo line sources)
onroad—high traffic segments—diesel	model as individual volume sources (pseudo line sources)

Table S3. List of pollutants and toxicity values used in the MNRiskS modeling system.

COPC Name	CAS #	RfD [mg/kg/day]	Oral CSF [mg/kg/day]	RFC [mg/m^3]	Inhalation URF [1/(µg/m^3)]
1,2,4TRIMETHYLBENZENE	95-63-6	0.05		0.007	
1,2-Epoxybutane	106-88-7			0.02	
1,3BUTADIENE	106-99-0		3.4	0.002	5.88E-05
2(2BUTOXYETHOXY)ETHANOL	112-34-5	0.03		0.0001	
2,2,4TRIMETHYLPENTANE	540-84-1				
2,4,4'-Trichlorobiphenyl	7012-37-5	5.00E-05	2	4.00E-07	0.00057
2,4D, SALTS AND ESTERS	94-75-7	0.01			
2BUTOXYETHYL ACETATE	112-07-2				
2CHLOROACETOPHENONE	532-27-4			3.00E-05	
2METHYLNAPHTHALENE	91-57-6	0.004			
2-Monochlorobiphenyl	2051-60-7	5.00E-05	2	4.00E-07	0.00057
2NITROPROPANE	79-46-9	0.00571	9.4	0.02	
3METHYLCHOLANTHRENE	56-49-5		22		0.0063
4,4METHYLENE DIANILINE	101-77-9	0.08	1.6	0.02	0.00046
4,4METHYLENEDIPHENYL DIISOCYANATE	101-68-8			0.0006	
5METHYL CHRYSENE	3697-24-3		2.8		0.0011
Acenaphthene	83-32-9	0.06			
ACENAPHTHYLENE	208-96-8				
Acetaldehyde	75-07-0			0.009	2.20E-06
ACETAMIDE	60-35-5		0.07		2.00E-05
Acetone	67-64-1	0.9			
Acetonitrile	75-05-8	0.006		0.06	
Acetophenone	98-86-2	0.1			
Acrolein	107-02-8	0.0005		0.0004	
ACRYLAMIDE	79-06-1	0.002	0.5	0.006	0.0001
ACRYLIC ACID	79-10-7	0.5		0.001	
Acrylonitrile	107-13-1	0.001	0.54	0.002	6.80E-05
ALDEHYDES	E761379			8.00E-05	5.00E-06
ALLYL CHLORIDE	107-05-1	0.000286	0.021	0.001	6.00E-06
AMMONIA	7664-41-7			0.08	
Aniline	62-53-3	0.007	0.0057	0.001	1.60E-06
Anthracene	120-12-7	0.3			
Antimony	7440-36-0	0.0004		0.0002	
Arsenic	7440-38-2	3.50E-06	1.5	1.50E-05	0.0043
Atrazine	1912-24-9	0.035	0.222		
BENZ(BK)FL	E17133943		0.28		0.00011
Benzaldehyde	100-52-7	0.1		0.02	
Benzene	71-43-2	0.004	0.055	0.03	7.80E-06

Table S3. *Cont.*

COPC Name	CAS #	RfD [mg/kg/day]	Oral CSF [mg/kg/day]	RFC [mg/m^3]	Inhalation URF [1/(μg/m^3)]
Benzo(a)anthracene	56-55-3		0.28		0.00011
Benzo(a)pyrene	50-32-8		2.8		0.0011
Benzo(b)fluoranthene	205-99-2		0.28		0.00011
BENZO(E)PYRENE	192-97-2				
BENZO(G,H,I)FLUORANTHENE	203-12-3				
BENZO(G,H,I)PERYLENE	191-24-2				
BENZO(J)FLUO	205-82-3		0.28		0.00011
Benzo(k)fluoranthene	207-08-9		0.28		0.00011
BENZOFLUORANTHENES	56832-73-6		0.28		0.00011
Benzyl chloride	100-44-7	0.002	0.17	0.001	4.90E-05
Beryllium	7440-41-7	0.002	8.4	2.00E-05	0.0024
BIPHENYL	92-52-4	0.05			
Bis(2-chlorethyl)ether	111-44-4		1.1		0.00033
Bromoform (Tribromomethane)	75-25-2	0.02	0.00793		1.10E-06
BUTYL CARBITOL ACETATE	124-17-4				
Butylbenzylphthalate	85-68-7	0.2	0.0019		
BUTYRALDEHYDE	123-72-8			0.07	
Cadmium	7440-43-9	0.001		2.00E-05	0.0018
CARBITOL	111-90-0	2			
CARBITOL ACETATE	112-15-2				
Carbon disulfide	75-15-0	0.1		0.7	
CARBON MONOXIDE	630-08-0				
Carbon tetrachloride	56-23-5	0.004	0.07	0.1	6.00E-06
CARBONYL SULFIDE	463-58-1				
CATECHOL	120-80-9		0.009		
CELLOSOLVE	110-80-5	0.4		0.2	
CELLOSOLVE ACETATE	111-15-9	0.3		0.3	
Chlorine	7782-50-5	0.1		0.0002	
CHLOROACETIC ACID	79-11-8	0.002			
Chlorobenzene	108-90-7	0.02		1	
Chloroethane	75-00-3			10	
Chloroform (Trichloromethane)	67-66-3	0.01	0.032	0.3	2.30E-05
Chloronaphthalene,2-	91-58-7	0.08			
CHLOROPRENE	126-99-8	0.02		0.02	0.0003
CHROM6 CMP	E5242631				
Chromium	7440-47-3				
CHROMIUM (III)	16065-83-1	1.5			
Chromium, hexavalent	18540-29-9	0.003	0.5	8.00E-06	0.012
Chrysene	218-01-9		0.028		1.10E-05

Table S3. *Cont.*

COPC Name	CAS #	RfD [mg/kg/day]	Oral CSF [mg/kg/day]	RFC [mg/m^3]	Inhalation URF [1/(µg/m^3)]
COBALT	7440-48-4	0.0003		6.00E-06	0.009
COPPER	7440-50-8	0.037			
CRESOL MIXED ISOMERS	1319-77-3	0.005		0.6	
Cresol, m-	108-39-4	0.05		0.6	
Cresol, o-	95-48-7	0.05		0.6	
Cresol, p-	106-44-5	0.005		0.6	
CROTONALDEHYDE	123-73-9	0.001	1.9		
Cumene (Isopropylbenzene)	98-82-8	0.1		0.4	
CYANIDE CMP	57-12-5	0.0006		0.003	
Dibenz(a,h)anthracene	53-70-3		4.1		0.0012
DIBENZ(AE)PY	192-65-4		1.1		0.0011
DIBENZ(AH)PY	189-64-0		2.5		0.011
DIBENZ(AI)PY	189-55-9		1.7		0.011
DIBENZ(AJ)AC	224-42-0		0.3		0.00011
DIBENZ(AL)PY	191-30-0		84		0.011
DIBENZOFURAN	132-64-9	0.001			
Dibromo-3-chloropropane, 1,2-	96-12-8	0.00057	7	0.0002	0.0019
Dichlorobenzene, 1,2-	95-50-1	0.09		0.2	
Dichlorobenzene, 1,3-	541-73-1				
Dichlorobenzene,1,4-	106-46-7	0.07	0.0054	0.8	1.10E-05
DICHLOROBENZENES	25321-22-6	0.09	0.024	0.8	1.10E-05
Dichloroethane 1,1-	75-34-3	0.2	0.0057		1.60E-06
Dichloroethane, 1,2-(Ethylene Dichloride)	107-06-2	0.03	0.091	0.4	2.60E-05
Dichloroethylene 1,1-	75-35-4	0.05		0.2	
Dichloroethylene, cis-1,2-	156-59-2	0.011			
Dichloropropane, 1,2-	78-87-5	0.09	0.036	0.004	
Dichloropropene, 1,3- (cis)	542-75-6	0.03	0.1	0.02	4.00E-06
Dichlorvos	62-73-7	0.0005	0.29	0.0005	8.30E-05
DICLPROP,13C	10061-01-5	0.03	0.1	0.02	4.00E-06
DIETHANOLAMINE	111-42-2	0.02		0.003	
DIETHYL SULFATE	64-67-5		1.2		
Dimethyl phthalate	131-11-3	10			
DIMETHYL SULFATE	77-78-1				
DIMETHYLANIL	121-69-7	0.002			
DIMETHYLBENZ(A)ANTHRACENE	57-97-6		250		0.071
DIMETHYLFORMAMIDE, N,N	68-12-2	0.1		0.03	
Dimethyoxybenzidine, 3,3’-	119-90-4		0.014		4.00E-05
Di-n-butyl phthalate	84-74-2	0.1			

Table S3. *Cont.*

COPC Name	CAS #	RfD [mg/kg/day]	Oral CSF [mg/kg/day]	RFC [mg/m^3]	Inhalation URF [1/(µg/m^3)]
Dinitrophenol, 2,4-	51-28-5	0.002			
Dinitrotoluene, 2,4-	121-14-2	0.002	0.31		8.90E-05
Di-n-octylphthalate	117-84-0	0.01			
Dioxane, 1,4-	123-91-1	0.03	0.1	3	7.70E-06
DIOXN 2378EQ	E17000407	1.00E-08	1,400,000	4.00E-08	400
Epichlorohydrin (1-Chloro-2,3-epoxypropane)	106-89-8	0.006	0.0099	0.001	1.20E-06
ETHYL ACRYLATE	140-88-5	0.0137	0.048		
Ethylbenzene	100-41-4	0.016		1	2.50E-06
Ethylene Dibromide	106-93-4	0.009	2	0.009	0.00022
ETHYLENE GLYCOL	107-21-1	2		0.4	
Ethylene oxide	75-21-8	0.00143	1.02	0.03	8.80E-05
Ethylhexyl phthalate, bis-2-	117-81-7	0.02	0.014		2.40E-06
FINE MNRLFIB	E649533				
Fluoranthene	206-44-0	0.04			
Fluorene	86-73-7	0.04			
Formaldehyde	50-00-0	0.2		0.009	5.00E-06
GLYCOL ETHRS	E651141			0.02	
HeptaCDD, 1,2,3,4,6,7,8-	35822-46-9	7.00E-08	14000	4.00E-06	4
HeptaCDF, 1,2,3,4,6,7,8-	67562-39-4	7.00E-08	14000	4.00E-06	4
HeptaCDF, 1,2,3,4,7,8,9-	55673-89-7	7.00E-08	14000	4.00E-06	4
Heptachlorobiphenyls, Total	28655-71-2	0.00033	30	0.0013	0.008571429
HexaCDD, 1,2,3,4,7,8-	39227-28-6	7.00E-09	140000	4.00E-07	40
HexaCDD, 1,2,3,6,7,8-	57653-85-7	7.00E-09	6200	4.00E-07	40
HexaCDD, 1,2,3,7,8,9-	19408-74-3	7.00E-09	140000	4.00E-07	40
HexaCDF, 1,2,3,4,7,8-	70648-26-9	7.00E-09	140000	4.00E-07	40
HexaCDF, 1,2,3,6,7,8-	57117-44-9	7.00E-09	140000	4.00E-07	40
HexaCDF, 1,2,3,7,8,9-	72918-21-9	7.00E-09	140000	4.00E-07	40
HexaCDF, 2,3,4,6,7,8-	60851-34-5	7.00E-09	140000	4.00E-07	40
Hexachloro-1,3-butadiene (Perchlorobutadiene)	87-68-3	0.001	0.078		2.20E-05
Hexachlorobenzene	118-74-1	0.0008	1.6		0.00046
Hexachlorobiphenyls, Total	26601-64-9	3.30E-07	30000	1.30E-06	8.571428571
Hexachlorocyclopentadiene	77-47-4	0.006		0.0002	
Hexachloroethane (Perchloroethane)	67-72-1	0.001	0.014		4.00E-06
HEXAMETHYLENE1,6DIISOCYANATE	822-06-0			1.00E-05	
HEXYETHANOL2	112-25-4				
HYDRAZINE	302-01-2	5.71E-05	3	0.0002	0.0049

Table S3. *Cont.*

COPC Name	CAS #	RfD [mg/kg/day]	Oral CSF [mg/kg/day]	RFC [mg/m^3]	Inhalation URF [1/(μg/m^3)]
Hydrogen chloride	7647-01-0			0.02	
HYDROGEN FLUORIDE	7664-39-3	0.04		0.014	
HYDROQUINONE	123-31-9	0.04	0.056		
Indeno(1,2,3-cd) pyrene	193-39-5		0.28		0.00011
Isophorone	78-59-1	0.2	0.00095	2	
Lead	7439-92-1		0.0085	0.00015	1.20E-05
MALEIC ANHYDRIDE	108-31-6	0.1		0.0007	
MANGANESE	7439-96-5	0.14		0.0002	
Mercuric chloride	7487-94-7				
Mercury	7439-97-6	0.0003		0.0003	
METHANE	74-82-8				
Methanol	67-56-1	0.5		4	
Methyl bromide (Bromomethane)	74-83-9	0.0014		0.005	
METHYL CARBITOL	111-77-3				
Methyl Cellosolve Acetate	110-49-6	0.008		0.09	
Methyl chloride (Chloromethane)	74-87-3			0.09	
Methyl ethyl ketone (2-Butanone)	78-93-3	0.6		5	
METHYL HYDRAZINE	60-34-4	0.001	1.1		
METHYL IODIDE	74-88-4		2.9		
Methyl isobutyl ketone	108-10-1	0.08		3	
Methyl mercury	22967-92-6	0.0001		0.00035	
METHYL METHACRYLATE	80-62-6	1.4		0.7	
METHYL TERT BUTYL ETHER	1634-04-4	0.3		3	2.60E-07
METHYLANTHRACENES	26914-18-1				
Methylene chloride	75-09-2	0.006	0.002	0.6	4.70E-07
METHYLNAPHTHALENE	90-12-0				
Naphthalene	91-20-3	0.02	0.12	0.009	3.40E-05
NHEXANE	110-54-3	0.06		2	
NHEXYL CARBITOL	112-59-4				
Nickel	7440-02-0	0.011		1.40E-05	0.00048
Nitrobenzene	98-95-3	0.002		0.009	4.00E-05
Nitrophenol, 4-	100-02-7	0.008			
n-Nitrosodimethylamine	62-75-9	8.00E-06	51		0.014
OctaCDD, 1,2,3,4,6,7,8,9-	3268-87-9	2.33E-06	420	0.000133333	0.12
OctaCDF, 1,2,3,4,6,7,8,9-	39001-02-0	2.33E-06	420	0.000133333	0.12
PentaCDD, 1,2,3,7,8-	40321-76-4	7.00E-10	1,400,000	4.00E-08	400
PentaCDF, 1,2,3,7,8-	57117-41-6	2.33E-09	42,000	1.33E-06	12
PentaCDF, 2,3,4,7,8-	57117-31-4	2.33E-10	420,000	1.33E-07	120

Table S3. *Cont.*

COPC Name	CAS #	RfD [mg/kg/day]	Oral CSF [mg/kg/day]	RFC [mg/m^3]	Inhalation URF [1/(μg/m^3)]
Pentachlorobiphenyls, Total	25429-29-2	1.00E-07	100,000	4.00E-07	28.57142857
Pentachloronitrobenzene (PCNB)	82-68-8	0.003	0.26	0.011	7.40E-05
Pentachlorophenol	87-86-5	0.005	0.4		5.10E-06
PERYLENE	198-55-0				
Phenanthrene	85-01-8				
Phenol	108-95-2	0.3		0.2	
PHOSPHINE	7803-51-2	0.0003		0.0003	
PHOSPHORUS (YELLOW OR WHITE)	7723-14-0	2.00E-05			
Phthalic anhydride (1,2-Benzene dicarboxylic anhydride)	85-44-9	2		0.02	
PM10	E1647619				
PM2.5 DIESEL	MDPM25			0.005	0.0003
PM2.5	E1647635				
POLYCHLORINATED BIPHENYLS (PCBS)	1336-36-3	5.00E-05	2	4.00E-07	0.00057
PPHENYLENEDIAMINE	106-50-3	0.19			
PROPIONALDEHYDE	123-38-6	0.008		0.008	
PROPYL CELLOSOLVE	2807-30-9				
PROPYLENE OXIDE	75-56-9	0.00857	0.24	0.03	3.70E-06
Pyrene	129-00-0	0.03			
QUINONE	106-51-4				
Selenium	7782-49-2	0.005		0.02	
Styrene	100-42-5	0.2		1	
SULFUR DIOXIDE	9/5/7446				
TetraCDD, 2,3,7,8-	1746-01-6	7.00E-10	1400000	4.00E-08	400
TetraCDF, 2,3,7,8-	51207-31-9	7.00E-09	140000	4.00E-07	40
Tetrachlorobiphenyls, Total	26914-33-0	3.30E-05	300	0.00013	0.085714286
Tetrachloroethane, 1,1,2,2-	79-34-5	0.02	0.2		5.80E-05
Tetrachloroethylene (Perchloroethylene)	127-18-4	0.006	0.0021	0.1	5.00E-07
Toluene	108-88-3	0.08		0.4	
TOLUENE2,4DIISOCYANATE	584-84-9			7.00E-05	1.10E-05
Toluidine, o-	95-53-4		0.016		5.10E-05
TOTAL PAH	130498-29-2		0.504		5.50E-05
Trichlorobenzene, 1,2,4-	120-82-1	0.01	0.0036		
Trichloroethane, 1,1,1-	71-55-6	2		5	
Trichloroethane, 1,1,2-	79-00-5	0.004	0.057		1.60E-05
Trichloroethylene	79-01-6	0.0005	0.046	0.002	3.30E-06

Table S3. *Cont.*

COPC Name	CAS #	RfD [mg/kg/day]	Oral CSF [mg/kg/day]	RFC [mg/m^3]	Inhalation URF [1/(µg/m^3)]
Trichlorofluoromethane (Freon 11)	75-69-4	0.3			
Trichlorophenol, 2,4,5-	95-95-4	0.1		0.35	
Trichlorophenol, 2,4,6-	88-06-2	0.001	0.011		3.10E-06
TRICHLOROTRIFLUOROMETHANE (CFC113, R113)	76-13-1	30			
TRIETHYLAMINE	121-44-8	0.002		0.007	
TRIFLURALIN	1582-09-8	0.0075	0.0077		
TRIMETHYLBENZENE	25551-13-7				
Trimethylbenzene, 1,3,5-	108-67-8	0.05			
Vinyl Acetate	108-05-4	1		0.2	
Vinyl Chloride	75-01-4	0.003	1.5	0.1	8.80E-06
XYLENES (MIXED ISOMERS)	1330-20-7	0.2		0.1	

	Traffic Density	Hazard Index All Sources	Cancer All Sourcese	Hazard Index On-Road Sources	Cancer On-Road Sources	Hazard Index Point Sources	Cancer Point Sources	Black	Native American	Hispanic	Non-White	< High School	Rent > 30% Income	Rent < $700	Commute by Walk or Transit	< 150% Poverty Level	Population Density	< 10 years of age	White	Bachelor's Degree	Drove Alone Commute	Median HH Income	Home Ownership %	Median Home Value	Vehicles per HH
**Hazard Index All Sources**	0.6	1.0																							
**Cancer All Sources**	0.6	0.9	1.0																						
**Hazard Index On-Road Sources**	0.6	0.8	0.9	1.0																					
**Cancer On-Road Sources**	0.6	0.8	0.8	0.9	1.0																				
**Hazard Index Point Sources**	0.5	0.7	0.7	0.7	0.7	1.0																			
**Cancer Point Sources**	0.5	0.7	0.7	0.7	0.7	0.8	1.0																		
**Black**	0.3	0.4	0.4	0.4	0.4	0.4	0.4	1.0																	
**Native American**	0.0	-0.1	-0.1	-0.1	-0.1	-0.1	-0.1	0.0	1.0																
**Hispanic**	0.1	0.2	0.2	0.2	0.2	0.1	0.2	0.2		1.0															
**Non-White**	0.3	0.4	0.4	0.4	0.4	0.3	0.4	0.5	0.1	0.4	1.0														
**< High School**	-0.1	-0.1	-0.1	-0.1	-0.1	-0.1	-0.1	0.1	0.1	0.2	0.1	1.0													
**Rent > 30% Income**	0.3	0.2	0.2	0.2	0.2	0.2	0.2	0.3	0.1	0.2	0.3	0.2	1.0												
**Rent < $700**	0.1				-0.1	-0.1	-0.1	0.1	0.1	0.1	0.1	0.3	0.5	1.0											
**Commute by Walk or Transit**	0.2	0.3	0.3	0.2	0.2	0.2	0.2	0.2	0.0	0.1	0.2	0.1	0.2	0.2	1.0										
**< 150% Poverty Level**	0.1					0.0	0.0	0.2	0.2	0.1	0.2	0.4	0.4	0.5	0.2	1.0									
**Population Density**	0.6	0.6	0.7	0.7	0.6	0.5	0.5	0.4	-0.1	0.2	0.4	-0.1	0.3	0.1	0.3	0.1	1.0								
**< 10 years of age**								0.1		0.1	0.1	0.0		0.0	0.0	0.1	0.0	1.0							
**White**	-0.3	-0.4	-0.4	-0.4	-0.4	-0.3	-0.4	-0.5	-0.1	-0.4	-1.0	-0.1	-0.3	-0.1	-0.2	-0.2	-0.4	-0.1	1.0						
**Bachelor's Degree**	0.2	0.2	0.2	0.3	0.3	0.3	0.3	0.1	-0.1	0.0	0.0	-0.5	-0.1	-0.3	0.1	-0.3	0.2	0.0	0.0	1.0					
**Drove Alone Commute**	0.1	0.0	0.1	0.1	0.1	0.0	0.1		-0.1			-0.3	-0.1	-0.2	-0.1	-0.2	0.1	0.1		0.2	1.0				
**Median HH Income**	0.0		0.0	0.1	0.1	0.1	0.1	-0.1	-0.2	-0.1	-0.1	-0.4	-0.4	-0.5	-0.2	-0.6		0.0	0.1	0.4	0.3	1.0			
**Home Ownership %**	-0.3	-0.2	-0.2	-0.2	-0.2	-0.1	-0.1	-0.3	-0.1	-0.2	-0.3	-0.2	-0.7	-0.6	-0.3	-0.4	-0.2		0.3	0.1	0.1	0.5	1.0		
**Median Home Value**	0.1	0.2	0.2	0.2	0.3	0.2	0.2	0.1	-0.1	0.0		-0.4	-0.2	-0.4	0.0	-0.4	0.1			0.4	0.2	0.5	0.2	1.0	
**Vehicles per HH**	-0.4	-0.4	-0.4	-0.4	-0.3	-0.3	-0.3	-0.3	0.0	-0.2	-0.3	-0.1	-0.5	-0.4	-0.3	-0.3	-0.4	0.0	0.3		0.2	0.4	0.5	0.1	1.0
**House Value > $250K**	-0.1	0.0	0.0				0.0	-0.1	-0.1	-0.1	-0.1	-0.4	-0.4	-0.4	-0.1	-0.5	-0.1		0.1	0.4	0.1	0.6	0.4	0.6	0.3

positive correlation $p < 0.05$

negative correlation $p < 0.05$

Figure S2. Kendall's Tau-b correlation matrix for all of Minnesota including both the metro and non-metro areas.

	Traffic Density	Hazard Index All Sources	Cancer All Sourcese	Hazard Index On-Road Sources	Cancer On-Road Sources	Hazard Index Point Sources	Cancer Point Sources	Black	Native American	Hispanic	Non-White	< High School	Rent > 30% Income	Rent < $700	Commute by Walk or Transit	< 150% Poverty Level	Population Density	< 10 years of age	White	Bachelor's Degree
**Hazard Index All Sources**	0.5	1.0																		
**Cancer All Sources**	0.6	0.8	1.0																	
**Hazard Index On-Road Sources**	0.7	0.7	0.7	1.0																
**Cancer On-Road Sources**	0.6	0.7	0.7	0.9	1.0															
**Hazard Index Point Sources**	0.4	0.5	0.5	0.5	0.5	1.0														
**Cancer Point Sources**	0.4	0.5	0.5	0.5	0.5	0.8	1.0													
**Black**	0.1	0.1	0.1	0.1	0.1	0.1	0.1	1.0												
**Native American**	0.0	-0.1	-0.1	-0.1	-0.1	-0.1	-0.1	0.1	1.0											
**Hispanic**	0.1		0.0					0.1		1.0										
**Non-White**	0.2	0.1	0.1	0.1	0.1	0.1	0.1	0.3	0.2	0.5	1.0									
**< High School**	-0.1	-0.1	-0.1	-0.1	-0.1	-0.1	-0.1	0.1	0.1	0.2	0.1	1.0								
**Rent > 30% Income**	0.3	0.2	0.3	0.3	0.3	0.1	0.1	0.2	0.1	0.1	0.2	0.2	1.0							
**Rent < $700**	0.3	0.1	0.2	0.2	0.1	0.1	0.1	0.1	0.1	0.1	0.2	0.2	0.6	1.0						
**Commute by Walk or Transit**	0.1	0.1	0.1					0.1		0.1	0.1	0.1	0.2	0.2	1.0					
**< 150% Poverty Level**	0.1	0.1	0.0	0.1	0.0			0.1	0.2	0.1	0.2	0.3	0.4	0.4	0.2	1.0				
**Population Density**	0.7	0.6	0.6	0.7	0.6	0.4	0.4	0.2	-0.1	0.1	0.2		0.4	0.3	0.1	0.1	1.0			
**< 10 years of age**			0.1	0.0	0.0					0.1	0.1						0.1	1.0		
**White**	-0.2	-0.1	-0.1	-0.1	-0.1	-0.1	-0.1	-0.3	-0.2	-0.5	-1.0	-0.1	-0.2	-0.2	-0.1	-0.2	-0.2	-0.1	1.0	
**Bachelor's Degree**	0.1	0.1	0.1	0.1	0.1	0.1	0.1		-0.1	-0.1		-0.4	-0.1	-0.1		-0.2	0.1	0.0	0.0	1.0
**Drove Alone Commute**	0.2	0.2	0.2	0.2	0.2	0.2	0.2		-0.1			-0.2	-0.1	-0.1	-0.1	-0.2	0.2	0.2		0.2
**Median HH Income**	-0.1						0.1	-0.1	-0.1	-0.1	-0.2	-0.4	-0.4	-0.5	-0.2	-0.6	-0.1	0.1	0.2	0.3
**Home Ownership %**	-0.3	-0.2	-0.3	-0.2	-0.2	-0.1	-0.1	-0.1	-0.1	-0.1	-0.2	-0.2	-0.7	-0.7	-0.2	-0.4	-0.3		0.2	0.1
**Median Home Value**	-0.1				0.1		0.1		-0.1	-0.1	-0.1	-0.3	-0.2	-0.3	-0.2	-0.4	-0.1		0.1	0.2
**Vehicles per HH**	-0.4	-0.3	-0.3	-0.3	-0.3	-0.2	-0.2	-0.1	0.0	-0.1	-0.2	-0.2	-0.5	-0.5	-0.2	-0.4	-0.5		0.2	
**House Value > $250K**	-0.3	-0.2	-0.2	-0.2	-0.2	-0.1	-0.1	-0.1		-0.1	-0.2	-0.2	-0.4	-0.4	-0.2	-0.4	-0.3		0.2	0.2

positive correlation $p < 0.05$

negative correlation $p < 0.05$

Figure S3. Kendall's Tau-b correlation matrix for the non-metro areas of Minnesota.

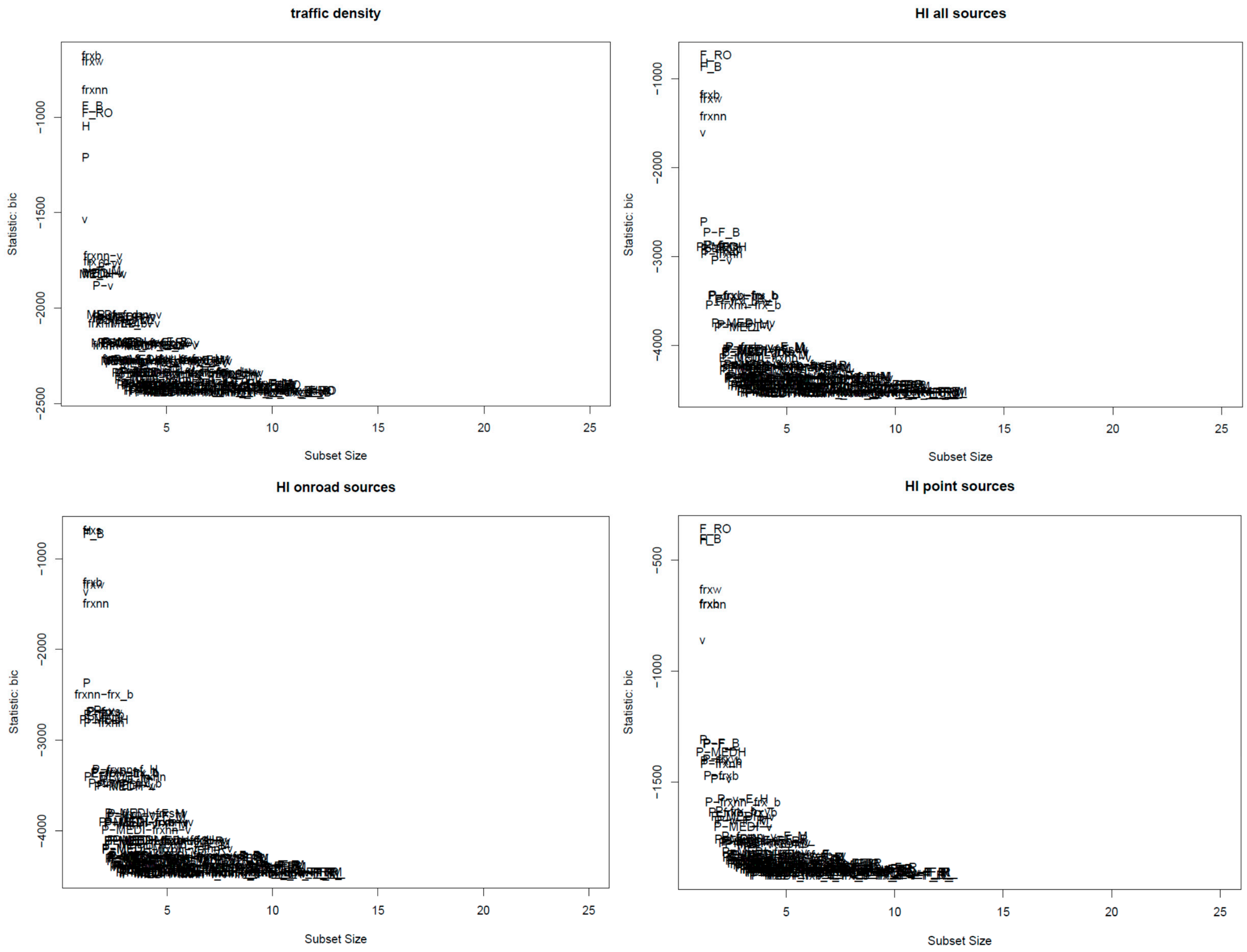

Figure S4. Regsubsets output showing the decrease (improvement) in the Bayesian Information Criterion as additional variables are added to the models relating traffic density, hazard index from all sources of air pollution, hazard index from on-road mobile sources of air pollution, and hazard index from point sources of air pollution to demographic variables.

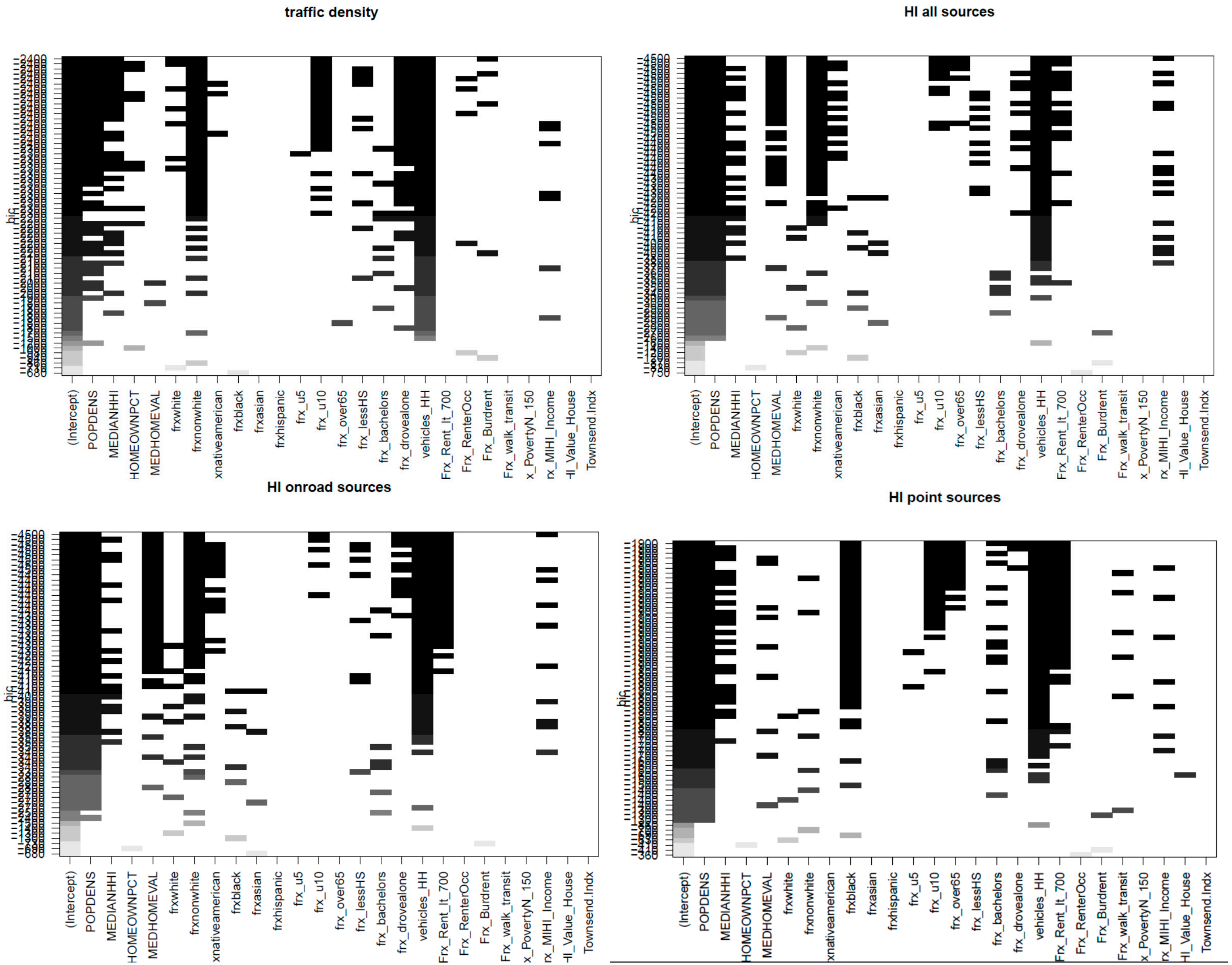

Figure S5. Regsubsets output showing the Bayesian Information Criterion for subsets of models relating demographic variables to traffic density, hazard index from all sources of air pollution, hazard index from on-road mobile sources of air pollution, and hazard index from point sources of air pollution.

Dependent Variable >>> Independent Variables vvv	Traffic Density	All Sources	Area Sources	Non-Road Sources	Onroad Sources	Point Sources	All Sources	Area Sources	Non-Road Sources	Onroad Sources	Point Sources
		Non-Cancer Hazard Index					Cancer Risk				
Non-white	0.18	0.25	0.32	0.15	-0.08	0.32	0.31	0.30	0.31	0.18	0.06
Commute by walk/transit			0.08	0.08	0.08	0.09	0.16	-0.03		0.04	
Population Density	0.27	0.54	0.42	0.58	0.65	0.39	0.21	0.52	0.42	0.42	0.16
Age less than 10 years	-0.10	-0.08	-0.09	-0.05		-0.10	-0.08	-0.09	-0.08	-0.09	
House value > $250K	0.11	0.23	0.17	0.03	-0.09	0.18	0.10	0.27	0.22	0.16	
Vehicles per Household	-0.42	-0.33	-0.28	-0.28	-0.20	-0.29	-0.25	-0.30	-0.24	-0.24	-0.10
R^2	0.40	0.60	0.56	0.60	0.45	0.56	0.40	0.58	0.46	0.35	0.05

Dependent Variable >>> Independent Variables vvv	Traffic Density	All Sources	Area Sources	Non-Road Sources	Onroad Sources	Point Sources	All Sources	Area Sources	Non-Road Sources	Onroad Sources	Point Sources
		Non-Cancer Hazard Index					Cancer Risk				
Non-white	-0.01	-0.12	-0.07	-0.12		-0.04		-0.06	-0.05		-0.07
Commute by walk/transit	-0.10	-0.09	-0.06	-0.06	-0.03	-0.06	-0.04	-0.08	-0.07	-0.06	
Population Density	0.90	1.01	1.08	1.21	1.11	0.96	0.85	1.00	0.92	0.12	
Age less than 10 years				0.03						-0.05	
House value > $250K		0.06	0.05	0.08	0.06	0.07		0.07	0.07	-0.09	
Vehicles per Household	-0.36	-0.28	-0.23	-0.23	-0.16	-0.27	-0.23	-0.25	-0.19	-0.13	-0.08
R^2	0.02	0.02	0.01	0.02	0.02	0.01	0.01	0.01	0.01	0.01	0.00

Figure S6. Results of stepwise linear regressions with log-transformed Non-Metro Area (top) and statewide (bottom) data. Each column represents one regression equation with the dependent variable listed at the top of the column and the independent variables defined in column one. The numbers in the body are the regression coefficients for the variables that were significant ($p < 0.05$) in the model. A blank means that the variable did not reach the significance level ($p < 0.05$) to be included in the model. The coefficients of determination (R2) are in the bottom row. Positive coefficients are highlighted in green and negative coefficients in red.

	Traffic Density			Non-Cancer All Sources			Cancer All Sources			Non-Cancer Area Sources		
	2.5%	coef	97.5%	2.5%	coef	97.5%	2.5%	coef	97.5%	2.5%	coef	97.5%
Non-white	0.10	0.15	0.20	0.18	0.22	0.26	0.20	0.25	0.29	0.17	0.21	0.25
Commute by walk/transit	0.04	0.10	0.16	0.13	0.18	0.22	0.18	0.23	0.28	0.16	0.20	0.25
Population Density	-0.09		0.03	0.24	0.29	0.33	0.10	0.15	0.20	0.30	0.35	0.39
Age less than 10 years	-0.18	-0.14	-0.10	-0.15	-0.12	-0.09	-0.12	-0.09	-0.05	-0.13	-0.10	-0.07
House value > $250K	-0.12	-0.07	-0.02	-0.15	-0.12	-0.08	-0.11	-0.07	-0.02	-0.14	-0.10	-0.06
Vehicles per Household	-0.39	-0.28	-0.27	-0.34	-0.30	-0.26	-0.34	-0.29	-0.25	-0.29	-0.25	-0.21

	Cancer Area Sources			Non-Cancer Nonroad			Cancer Nonroad			Non-Cancer Onroad		
	2.5%	coef	97.5%	2.5%	coef	97.5%	2.5%	coef	97.5%	2.5%	coef	97.5%
Non-white	0.07	0.10	0.13	0.21	0.25	0.30	0.21	0.26	0.31	0.21	0.20	0.30
Commute by walk/transit	0.10	0.13	0.17	0.23	0.29	0.33	0.26	0.32	0.37	0.23	0.09	0.33
Population Density	0.63	0.66	0.69	0.05	0.10	0.14	-0.08		0.03	0.05	0.27	0.14
Age less than 10 years	-0.09	-0.07	-0.04	-0.14	-0.11	-0.07	-0.12	-0.09	-0.05	-0.14	-0.11	-0.07
House value > $250K	-0.09	-0.07	-0.04	-0.13	-0.09	-0.05	-0.08		0.01	-0.13	-0.12	-0.05
Vehicles per Household	-0.23	-0.20	-0.17	-0.32	-0.27	-0.22	-0.29	-0.24	-0.19	-0.32	-0.28	-0.22

	Cancer Onroad			Non-Cancer Point Sources			Cancer Point Sources		
	2.5%	coef	97.5%	2.5%	coef	97.5%	2.5%	coef	97.5%
Non-white	0.13	0.18	0.23	0.07	0.12	0.17	-0.03		0.09
Commute by walk/transit	0.04	0.10	0.16	0.21	0.26	0.32	-0.02		0.12
Population Density	0.12	0.18	0.24	0.24	0.30	0.35	0.03	0.09	0.16
Age less than 10 years	-0.11	-0.07	-0.03	-0.12	-0.09	-0.05	-0.08		0.02
House value > $250K	-0.11	-0.06	-0.02	-0.05		0.04	-0.21	-0.15	-0.09
Vehicles per Household	-0.32	-0.26	-0.21	-0.31	-0.26	-0.20	-0.08		0.05

Figure S7. Confidence intervals for coefficients in the final regression models.

	Traffic density	Cancer Risk					Non-Cancer Hazard Index				
		on-road sources	non-road sources	area sources	point sources	all sources	on-road sources	non-road sources	area sources	point sources	all sources
Population Average	1.77	2.2E-04	1.3E-04	4.8E-05	5.3E-06	4.0E-04	0.60	0.36	0.47	0.23	1.66
Asian	2.95	3.9E-04	2.6E-04	6.6E-05	7.8E-06	7.2E-04	1.01	0.68	0.74	0.34	2.78
Black	4.30	4.9E-04	3.2E-04	8.4E-05	9.0E-06	9.0E-04	1.28	0.83	0.98	0.51	3.61
Hispanic	2.75	3.3E-04	2.1E-04	6.2E-05	7.9E-06	6.1E-04	0.86	0.56	0.68	0.35	2.46
American Indian	1.66	1.7E-04	1.3E-04	4.9E-05	3.8E-06	3.6E-04	0.48	0.34	0.50	0.21	1.52
NonWhite	3.14	3.8E-04	2.5E-04	6.8E-05	7.8E-06	7.0E-04	0.99	0.65	0.77	0.39	2.80
White	1.51	1.9E-04	1.1E-04	4.4E-05	4.8E-06	3.5E-04	0.52	0.31	0.41	0.21	1.44
Less than High School	2.13	2.3E-04	1.6E-04	5.4E-05	5.4E-06	4.6E-04	0.64	0.42	0.55	0.26	1.87
Bachelors Degree	1.92	2.5E-04	1.4E-04	4.8E-05	5.6E-06	4.4E-04	0.67	0.39	0.48	0.27	1.81
House Value > $250K	1.39	1.1E-04	1.1E-04	3.7E-05	4.8E-06	2.6E-04	0.56	0.31	0.36	0.21	1.44
Owner Occupied Housing	1.42	1.9E-04	1.1E-04	4.2E-05	4.9E-06	3.4E-04	0.52	0.30	0.39	0.21	1.42
Rent < $700	3.39	2.9E-04	2.2E-04	8.2E-05	6.7E-06	5.9E-04	0.80	0.55	0.79	0.34	2.48
Rent > 30% Income	3.58	3.5E-04	2.4E-04	7.9E-05	7.3E-06	6.8E-04	0.95	0.63	0.83	0.39	2.80
Rental Housing	3.34	3.3E-04	2.2E-04	7.4E-05	7.1E-06	6.3E-04	0.89	0.58	0.77	0.36	2.60
>1 Vehicles in Household	1.43	1.9E-04	1.1E-04	4.2E-05	4.7E-06	3.4E-04	0.51	0.30	0.39	0.20	1.41
Commute by Walk/Transit	3.26	3.4E-04	2.5E-04	7.5E-05	7.4E-06	6.7E-04	0.92	0.64	0.82	0.41	2.80
Drove Alone	1.66	2.1E-04	1.2E-04	4.5E-05	5.2E-06	3.8E-04	0.58	0.34	0.44	0.22	1.57
No Vehicles in Household	4.03	3.7E-04	2.6E-04	8.6E-05	7.6E-06	7.3E-04	0.99	0.68	0.89	0.42	2.98
Below 100% of Poverty	2.84	2.9E-04	2.2E-04	7.1E-05	6.3E-06	5.9E-04	0.80	0.56	0.74	0.33	2.42
Below 150% of Poverty	2.59	2.7E-04	1.9E-04	6.5E-05	6.1E-06	5.3E-04	0.74	0.50	0.67	0.30	2.21
HH Income > $60K	1.60	2.1E-04	1.2E-04	4.3E-05	5.3E-06	3.8E-04	0.59	0.34	0.42	0.23	1.57
Children Under 10	1.66	2.2E-04	1.3E-04	4.7E-05	5.2E-06	4.0E-04	0.59	0.35	0.46	0.22	1.62
Over 65	1.63	1.9E-04	1.1E-04	4.4E-05	4.7E-06	3.5E-04	0.52	0.30	0.41	0.21	1.44

Figure S8. Breakdown of the Statewide traffic density, cancer risks, and non-cancer hazard indices by demographic group and pollution source category.

© 2015 by the authors; licensee MDPI, Basel, Switzerland. This article is an open access article distributed under the terms and conditions of the Creative Commons Attribution license (http://creativecommons.org/licenses/by/4.0/).
